# Impact of rapid enterovirus polymerase chain reaction testing on management of febrile young infants < 90 days of age with aseptic meningitis

**DOI:** 10.1186/s12887-020-02066-0

**Published:** 2020-04-16

**Authors:** Paolo Paioni, Florence Barbey, Christa Relly, Patrick Meyer Sauteur, Christoph Berger

**Affiliations:** Division of Infectious Diseases and Hospital Epidemiology, University Children’s Hospital Zurich, Steinwiesstrasse 75, CH-8032 Zürich, Switzerland

**Keywords:** Children, Fever, Sepsis, Pleocytosis, Antibiotic treatment

## Abstract

**Background:**

Diagnostic evaluation of febrile young infants is challenging. Empirical antimicrobial treatment is therefore common practice in this setting despite high percentage of causative viral infections. The objective of this study was to investigate the impact of rapid enterovirus cerebrospinal fluid polymerase chain reaction (CSF EV PCR) test on hospital length of stay (LOS) and antimicrobial treatment duration in young febrile infants.

**Methods:**

Retrospective observational study comparing duration of antimicrobial treatment and hospital LOS before (May 1, 2014 - May 30, 2015, untested group) and after (June 1, 2015 - June 30, 2017, tested group) the introduction of rapid CSF EV PCR testing in infants < 90 days of age presenting with fever and CSF pleocytosis at the University Children’s Hospital Zurich. Additionally, the same variables were compared after test introduction between CSF EV PCR positive and negative children.

**Results:**

One hundred twenty-eight children were enrolled in the study, 58 before and 70 after the introduction of rapid CSF EV PCR testing. Duration of antimicrobial treatment was significantly shortened in EV positive (*n* = 42) compared to both EV negative (*n* = 28) (median 18 h and 48 h, respectively, *p* < 0.001) and untested patients (*n* = 58) (median 18 h and 48 h, respectively, *p* < 0.001), and also in tested compared to untested group patients (median 36 vs 48 h, p < 0.001). Hospital LOS was significantly shortened in EV positive compared to EV negative patients (median 3 days and 4 days respectively, *p* = 0.013), while an overall reduction was not observed between tested and untested group patients.

**Conclusions:**

In this study we demonstrate that antimicrobial treatment duration could be significantly shortened in neonates and young infants < 90 days of age with aseptic meningitis after the introduction of a rapid CSF EV PCR test compared to untested patients before test introduction.

## Background

The diagnostic evaluation of febrile young infants (< 90 days of age) is challenging. Clinical and laboratory findings at presentation do not allow to reliably identify and treat only those with serious bacterial infections [1–3]. Neonates and young infants with fever are therefore frequently hospitalized and empirically treated with parenteral broad-spectrum antibiotics until bacterial culture results are available [2, 4]. However, most of these patients suffer from viral infections [2]. Enteroviruses (EV) are found in 25–60% of febrile infants < 90 days of age and are the main cause of aseptic meningitis, not only in summer and fall but even during offseason [5–8]. Nevertheless, the diagnosis of EV meningitis remains challenging: first, because cerebrospinal fluid (CSF) pleocytosis and polymorphonuclear cells predominance in CSF do not allow to certainly discriminate between viral and bacterial etiology, and second, because CSF pleocytosis may be absent despite EV meningitis in up to 34% of febrile infants < 90 days of age and in up to 77% of febrile neonates [6, 9–12]. Rapid detection of EV in CSF by reverse transcription polymerase chain reaction (PCR) allows accurate and rapid diagnosis of infants with EV meningitis. CSF EV PCR testing has previously been shown to decrease antimicrobial treatment duration and hospital length of stay (LOS) in infants with aseptic meningitis [4, 6, 10, 13–17]. However, these previous studies investigated the impact of CSF EV PCR tests with results available after 12 h and longer [6, 10, 14, 18], at any age [10, 13, 15–17, 19–21], and/or restricted the study period to summer and fall [15, 20]. Additionally patients were exclusively compared depending on CSF EV PCR results [6, 10, 13, 15], before and after the introduction of the test [17], or without a proper control group [18–20].

The objective of this study was to investigate the impact of a rapid CSF EV PCR with time to result of 3 to 6 h on hospital LOS and duration of antimicrobial treatment in children < 90 days of age with aseptic meningitis.

## Methods

### Study design

We performed a retrospective observational study at the University Children’s Hospital of Zurich. Demographic, clinical, and laboratory data including hospital LOS and duration of antimicrobial treatment were extracted from medical records and the microbiology laboratory database.

### Patients

Previously healthy infants < 90 days of age (i.e. children without any underlying condition) presenting at our emergency department with fever (rectal temperature ≥ 38.0 °C) were eligible for inclusion if they underwent standardized full sepsis evaluation (consisting of analysis of blood, catheter urine and CSF) prior to antimicrobial treatment between May 1, 2014 and June 30, 2017. In house rapid CSF EV PCR testing was introduced June 1, 2015. The test was not routinely applied to all febrile infants under the age of 90 days. The attending physician decided whether to perform the test based mainly on exposure history, seasonality and the presence of CSF pleocytosis. Infants presenting with fever and CSF pleocytosis, defined as the presence of ≥5 white blood cells (WBC) per μl, after test introduction (June 1, 2015 - June 30, 2017; 25 months) were included in the tested group if they had CSF EV PCR performed at admission, and those presenting within 13 months before its introduction (May 1, 2014 - May 30, 2015; 13 months) in the untested group. Although the reported thresholds for definition of CSF pleocytosis in children < 8 weeks may be higher [22, 23], the definition of 5 WBC per μl CSF was used as threshold for CSF EV PCR testing at our institution in order to avoid missing neonates with low or absent CSF pleocytosis despite enteroviral meningitis as described in previous studies [6, 9–12]. Since no bacterial infections such as bacteremia, bacterial meningitis or urinary tract infection (UTI) were observed in the tested group patients, children with bacterial infections were excluded also from the untested group to prevent bias due to prolonged hospital LOS and antimicrobial treatment associated with these conditions. Children with bacterial contamination in blood, urine or CSF culture on the other hand were not excluded from the analysis. Bacterial contamination was defined as identification of a common skin commensal (i.e. coagulase-negative staphylococci including *S. epidermidis*, viridans group streptococci, *Bacillus* spp., *Propionibacterium* spp., *Corynebacterium* spp., *Aerococcus* spp. and *Micrococcus* spp.) in blood or CSF cultures or growth of 2 or more bacteria in urine culture.

### CSF EV PCR testing

CSF EV PCR was performed using the commercially available Xpert® EV method (Cepheid, Sunnyvale, California, USA). The assay runs on GeneXpert® system with integrated sample processing, viral RNA extraction, reverse transcription (RT) and nucleic acid amplification, as well as detection of the target sequence by real-time PCR, in a single reaction cartridge. The following EV species and subtypes are detected: Species A (Coxsackie A2-A8, A10, A12, A14, A16, EV71), Species B (Coxsackie A9, B1-B6, Echo 1–7, 9, 11–21, 24–27, 29–33, EV69), Species C (Coxsackie A11, A13, A15, A17–22, A24), Species D (EV68, EV70) and Poliovirus (Poliovirus 1–3). The test has a running time of less than 2.5 h and was available every day between 07:00 AM and midnight in the in-house microbiology laboratory. During these 17 h, treating physicians were informed immediately about the test results with a time to result of 3 to 6 h. CSF samples awaiting processing were kept at 4 °C until testing.

### Data analysis

Continuous data were described using median and interquartile range (IQR), whereas categorical data were described using frequency and percentage. Mann-Whitney *U* test was used to compare medians between groups. For the comparison of more than two groups Pearson Chi-squared and Kruskal-Wallis tests were used for categorical and continuous data respectively. Subsequent post hoc testing by pairwise comparisons was performed in case of statistical significance in order to identify the groups with significant differences. All analyses were performed with IBM SPSS Statistics version 24. Two-tailed *p*-values smaller than 0.05 were considered to be statistically significant.

## Results

**Fig. 1:**
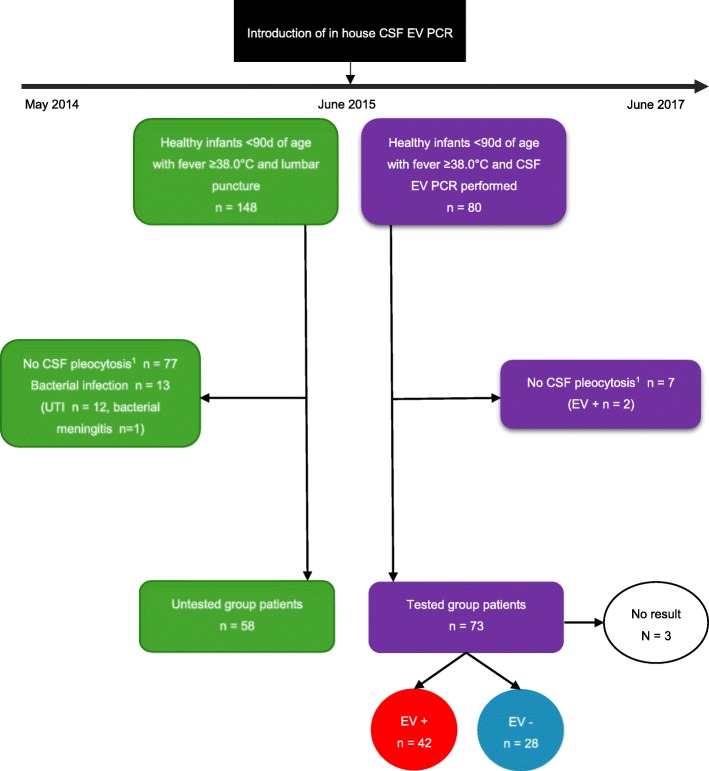
Study profile. Recruitment and flow of patients in untested group (left) and tested group (right). ^1^Pleocytosis defined as the presence of ≥5 white blood cells per μl in CSF. Abbreviations: EV, enterovirus; CSF, cerebrospinal fluid; PCR, polymerase chain reaction; UTI, urinary tract infection; d, days

During the three-year study period, a total of 228 previously healthy infants < 90 days of age presented with fever and underwent full sepsis evaluation (Fig. 1). After introducing CSF EV PCR testing, the test was performed in 80 febrile infants < 90 days. Thereof, 7 patients were excluded because CSF EV PCR was requested despite the lack of pleocytosis (≥5 cells/μl CSF) and in 3 cases the test failed and no result was available. Among the 70 patients included in the tested group, 42 (60%) were tested positive by CSF EV PCR and no bacterial co-infection (sepsis, bacterial meningitis, UTI) was documented. In the period before the introduction of CSF EV PCR testing, 148 febrile patients < 90 days underwent full sepsis evaluation. Thereof, 77 patients were excluded due to lack of CSF pleocytosis and 13 due to presence of bacterial infection. The remaining 58 patients were included in the untested group. Demographic characteristics and laboratory results of the patients are shown in Table 1. Of note, while age, C-reactive protein (CRP) and CSF glucose did not significantly differ in CSF EV PCR-positive compared to negative and untested group patients, a significantly higher level of CSF leucocytes was found for CSF EV PCR positive compared to negative patients (median 127 cells/μl and 17 cells/μl, respectively, *p* < 0.001).

**Table 1:** Patients characteristics

	Tested group		Untested group	
	CSF EV PCR +	CSF EV PCR -		*p*-value^1^
Patients, n	42	28	58	
Age in days, median (IQR)	35 (17–48)	39 (33–64)	38 (23–58)	0.133
Gender male, n (%)	24 (57.1%)	15 (53.6%)	34 (58.6%)	0.906
Blood WBC, G/l, median (IQR)	8.9 (7.5–11.2)	8.5 (6.6–11)	10.5 (7.8–13.3)	0.107
CRP, mg/l, median (IQR)	4 (4–13)	6 (4–22)	4 (4–11)	0.344
CSF WBC, cells/μl, median (IQR)	127 (32–496)	17 (10–60)	18 (8–63)	**< 0.011**^**2**^
Mononuclear in %, median (IQR)	80.1 (58.1–94)	85 (59.7–94)	87.1 (75–100)	**0.049**^**3**^
CSF protein, g/l, median (IQR)	0.69 (0.55–0.94)	0.65 (0.43–0.92)	0.58 (0.42 -0.71)	**0.031**^**3**^
CSF glucose, mmol/l, median (IQR)	2.7 (2.5–2.9)	2.8 (2.7–3.1)	2.8 (2.5–2.9)	0.559
Contamination rate overall, n (%)^4^	10 (23.8%)	10 (35.7%)	23 (39.7%)	0.245

Abbreviations: *CSF* cerebrospinal fluid, *EV* enterovirus, *WBC* white blood cells, *CRP* C-reactive protein, *IQR* interquartile range;

^1^ Pearson Chi-squared test for categorical data, Kruskal-Wallis test for continuous data

^2^ Statistically significant difference between CSF EV PCR + and – patients (*p* < 0.001) and between CSF EV PCR + and untested group patients (*p* = 0.011)

^3^ Statistically significant difference between CSF EV PCR + and untested group patients

^4^ Contamination defined as non-relevant bacterial growth in culture from blood, CSF and/or urine

**Fig. 2:**
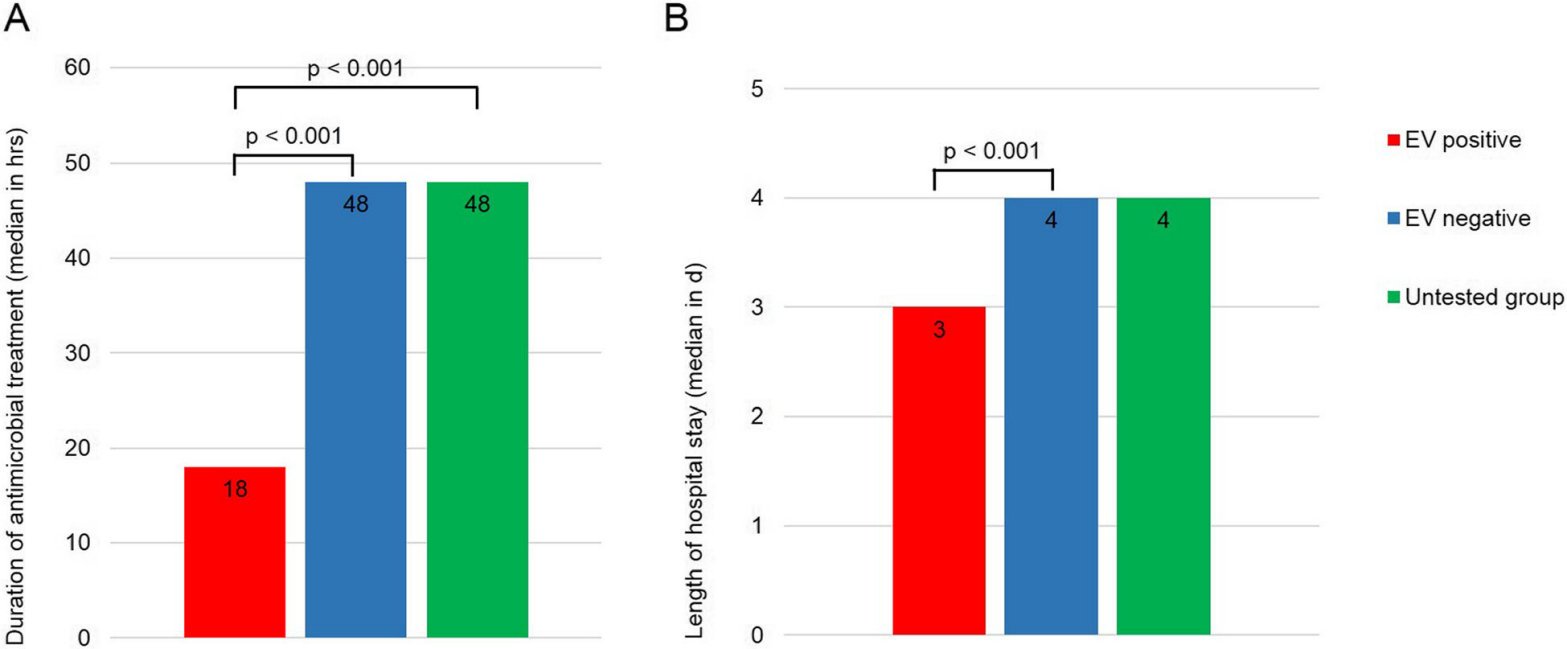
Median duration of antimicrobial treatment (**a**) and hospital LOS (**b**). Comparison of duration of antimicrobial treatment (**a**) and hospital LOS (**b**) between CSF EV CSF PCR positive, negative and untested group patients using Kruskal-Wallis test. The number in the bars indicates the median value. Abbreviations: LOS, length of stay; EV, enterovirus; CSF, cerebrospinal fluid; PCR, polymerase chain reaction; hrs, hours; d, days

**Fig. 3:**
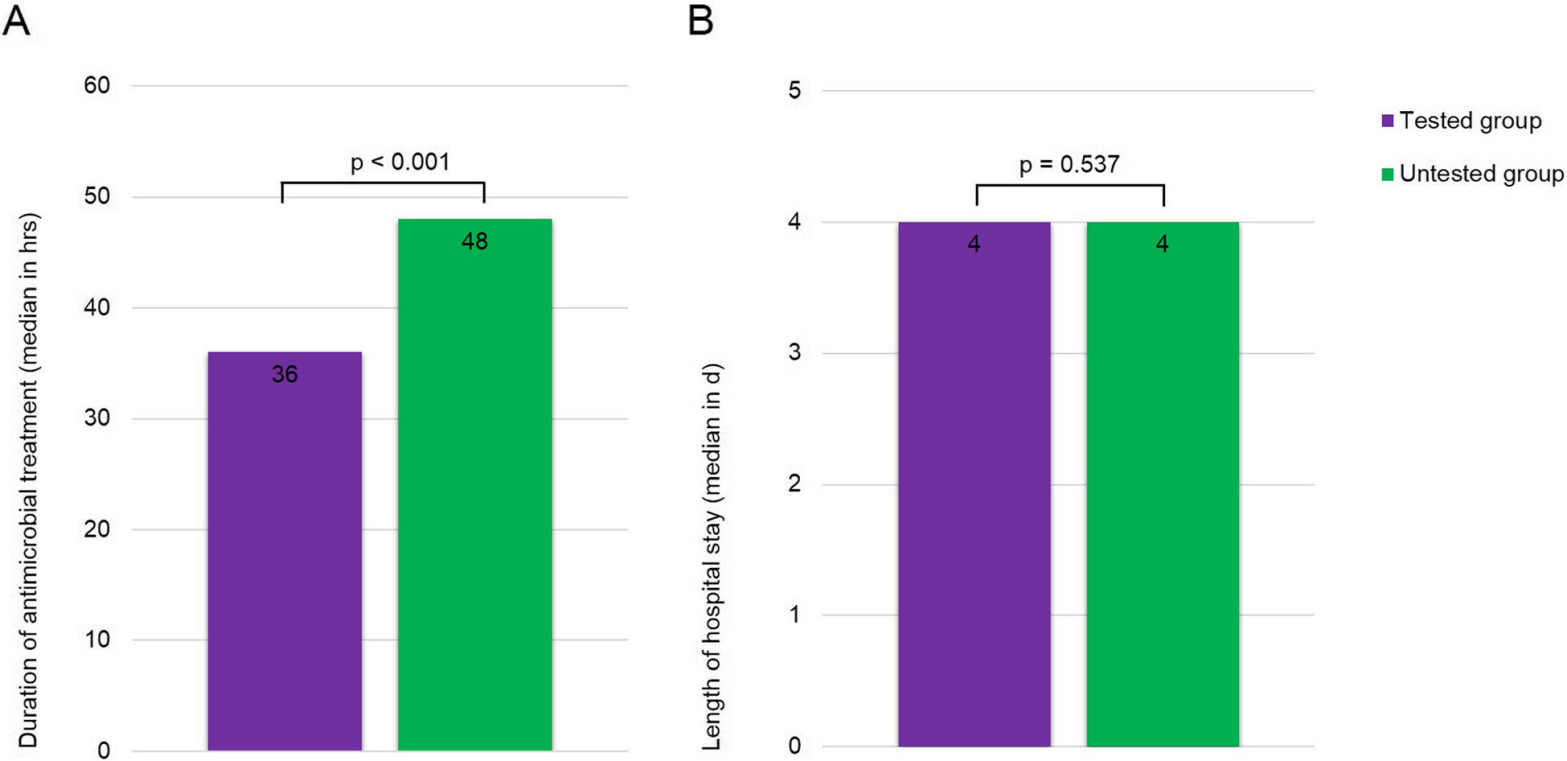
Comparison of duration of antimicrobial treatment (**a**) and hospital LOS (**b**) between tested and untested group patients. Comparison of medians between tested group (i.e. CSF EV CSF positive and negative patients) and untested group patients using the Mann-Whitney U test. The number in the bars indicates the median value. Abbreviations: LOS, length of stay; EV, enterovirus; CSF, cerebrospinal fluid; PCR, polymerase chain reaction; hrs, hours; d, days

Antimicrobial treatment duration was significantly shortened by more than half in CSF EV PCR-positive compared to both negative patients (median 18 h and 48 h, respectively, *p* < 0.001) and untested group patients (median 18 h and 48 h, respectively, *p* < 0.001) (Fig. 2a). Furthermore, the duration of antimicrobial treatment was significantly shorter in the tested compared to the untested group patients (median 36 vs 48 h, *p* < 0.001; Fig. 3a).

Hospital LOS was significantly shorter in CSF EV PCR positive compared to negative patients (median 3 days and 4 days, respectively, *p* < 0.001 as shown in Fig. 2b while an overall reduction was not observed between patients in the tested and untested group (4 days in both groups, *p* = 0.537; Fig. 3b).

Bacterial contamination rates detected in cultures from blood, CSF and urine did not differ between CSF EV PCR positive, negative and untested group patients (Table 1). The analysis of hospital LOS and duration of antimicrobial treatment showed similar results after exclusion of patients with contaminated bacterial cultures (median duration of antimicrobial treatment 24 h in the tested group vs. 48 h in the untested group, *p* < 0.001; median hospital LOS 4 days in both groups, *p* = 0.117).

## Discussion

In this observational study we demonstrate that antimicrobial treatment duration could be significantly shortened in neonates and young infants < 90 days of age with aseptic meningitis at our institution after the introduction of a rapid CSF EV PCR test compared to untested patients before test introduction. This is mostly due to the effect of the significant reduction of antimicrobial treatment duration by more than 60% to a median length of treatment of less than 24 h in EV positive compared to EV negative patients (18 vs 48 h, respectively). Additionally, a significant reduction of median hospital LOS by at least one day for febrile infants < 90 days of age tested CSF EV PCR positive was achieved compared to those tested negative (3 vs 4 days). These findings confirm previous studies reporting a reduction of antimicrobial treatment duration between 30 and 50% [4, 6, 10, 13, 15, 19]. The greater extent of the reported reduction of antimicrobial treatment duration in our analysis may be linked to the rapid availability of CSF EV PCR results. While the duration of the PCR in the microbiology laboratory takes 2.5 h, the time to result depends on the pre-analytical time (time from lumbar tap to start of the PCR run) and the post-analytical time (time between end of PCR run, interpretation and reporting of the result to the clinician). Such a short time to result of 3 to 6 h as in our study was only evaluated by Archimbaud et al. and Huizing et al. [16, 17] in CSF EV PCR positive infants before and after routine introduction of the test.

The majority of neonates and young children < 90 days of age presenting with fever are hospitalized in good general condition and empirically treated for potential sepsis, even if unlikely, awaiting negative culture results from blood, CSF and urine for 48–72 h [24]. As the initial clinical presentation does not allow to discriminate invasive bacterial from viral infections and enterovirus infections are frequent in this age group, identification of EV infection might allow to stop antimicrobial treatment without awaiting results from bacterial cultures and discharge these infants early, if in good condition. This approach is feasible in everyday practice as there is a very low risk of concomitant bacterial infection in CSF EV PCR positive patients in this and other reports [3, 17, 25]. Furthermore Nigrovic et al. [7] have shown that infants older than 4 weeks with EV meningitis have a low risk for bacterial co-infection and might be safely treated as outpatients, assuming they appear to be well and are followed up adequately.

In our study the effect of EV testing in CSF was not yet strong enough to demonstrate a significant reduction of hospital LOS before and after test introduction. Possible explanations for these findings are the not yet systematic introduction of the test (i.e. the attending physician decided whether or not to perform the test), the lack of rapid implementation (i.e. discharge of the patient by the responsible team of physicians at bedside as soon as the test result was available) and to a lesser extent the non-availability of the test during the night. It remains to be seen whether systematic testing, immediate reporting of the test result and its subsequent implementation with cessation of antimicrobial treatment in EV positive infants, if in good general condition and with normal urine analysis, may allow to achieve a significant overall reduction of hospital LOS.

Significantly higher CSF leucocyte levels have been described for CSF EV PCR positive patients [5] and could be confirmed in our study. King et al. [6] by stratifying patients according to presence or absence of CSF pleocytosis, demonstrated significant decreases of hospital LOS in both groups tested positive for CSF EV PCR. Nevertheless, the absence of CSF pleocytosis in neonates with enteroviral meningitis is a well-known phenomenon. The lack of CSF pleocytosis in 34–77% of neonates with enteroviral meningitis in previous studies [11, 12] does not allow to use CSF pleocytosis as an indicator for enteroviral meningitis in this age group. In fact applying the more restrictive reference values of liquor pleocytosis according to Bonadio et al. [23] (age < 4 weeks > 22 cells/μl CSF, age 4–7 weeks > 15 cells/μl CSF and age > 8 weeks > 5 cells/μl CSF) 8 (22%) of the EV positive patients in our study showed absence of CSF pleocytosis. Despite the low threshold for CSF pleocytosis (≥5 WBC per μl) used in this study, some young infants with EV meningitis might have been missed. In fact, 2 out of 7 patients excluded because of lack of pleocytosis (Fig. 1) tested CSF EV PCR positive.

Therefore, the fact that pleocytosis was the main indication to perform the test is a limitation of our study. Other limitations of our work are its retrospective design, the small sample size and the unsystematic use of the CSF EV PCR test in febrile infants < 90 days of age.

## Conclusions

The use of rapid CSF EV PCR testing in febrile infants < 90 days of age undergoing full sepsis evaluation leads to a significant reduction of antimicrobial treatment duration and hospital LOS in EV positive compared to negative patients but also to an overall reduction of antimicrobial treatment in patients with aseptic meningitis. These benefits of rapid CSF EV PCR testing in febrile neonates and infants on duration of treatment and hospital stay may be increased if systematic testing is introduced (e.g. testing all febrile infants < 90 days of age who undergo lumbar puncture during enteroviral season independently of pleocytosis), time to reporting of the test result is optimized, and medical staff is instructed to rapidly implement the positive test result on patient management.

## Data Availability

The datasets used and/or analysed during the current study are available from the corresponding author on reasonable request.

## Abbreviations

CRP: C-reactive protein
CSF: Cerebrospinal fluid
EV: Enterovirus
LOS: Length of stay
PCR: Polymerase chain reaction
UTI: Urinary tract infection
WBC: White blood cells
